# 2-(3,4-Dihydro-4-Oxothieno[2,3-*d*]pyrimidin-2-ylthio) Acetamides as a New Class of Falcipain-2 Inhibitors. 3. Design, Synthesis and Biological Evaluation

**DOI:** 10.3390/molecules14020785

**Published:** 2009-02-16

**Authors:** Jin Zhu, Tong Chen, Jie Liu, Ruoqun Ma, Weiqiang Lu, Jin Huang, Honglin Li, Jian Li, Hualiang Jiang

**Affiliations:** 1 School of Pharmacy, East China University of Science and Technology, 130 Mei Long Road, Shanghai 200237, P.R. China; 2 Drug Discovery and Design Center, Shanghai Institute of Materia Medica, Chinese Academy of Sciences, 555 Zu Chong Zhi Road, Shanghai 201203, P.R. China

**Keywords:** 2-(3,4-Dihydro-4-oxothieno[2,3-*d*]pyrimidin-2-ylthio)acetamide derivatives, Falcipain-2 inhibitor, Malaria, SAR

## Abstract

The cysteine protease falcipain-2 (FP-2) of *Plasmodium falciparum* is a principal cysteine protease and an essential hemoglobinase of erythrocytic *P. falciparum* trophozoites, making it become an attractive target enzyme for developing anti-malarial drugs. In this study, a series of novel small molecule FP-2 inhibitors have been designed and synthesized based on compound **1**, which was identified by using structure-based virtual screening in conjunction with an enzyme inhibition assay. All compounds showed high inhibitory effect against FP-2 with IC_50_s of 1.46-11.38 μM, and the inhibitory activity of compound **2a** was ~2 times greater than that of prototype compound **1**. The preliminary SARs are summarized and should be helpful for future inhibitor design, and the novel scaffold presented here, with its potent inhibitory activity against FP-2, also has potential application in discovery of new anti-malarial drugs.

## Introduction

Malaria remains one of the most important infectious disease problems in the world, accounting for 300-500 million clinical cases and up to 2.7 million deaths each year. About 90% of these casualties occur in tropical Africa, and the great majority are children under the age of 5 [1]. *Plasmodium falciparum*, one of the four species of *Plasmodium*, is the most lethal protozoan parasite of the genus, which is responsible for malaria. At present no effective vaccines are available due to the high mutability of the genome of *Plasmodium falciparum* [2], meanwhile, resistance of malaria parasites to available conventional drug therapy is an increasingly serious problem [3,4,5]. Accordingly, the discovery of new effective drugs to counter the spread of malaria parasites that are resistant to existing agents, especially acting on new targets, is an urgent need.

Among various potential new targets, the cysteine protease falcipain-2 (FP-2) of *P. falciparum* is an attractive and promising target enzyme [6,7]. FP-2 is a principal cysteine protease and essential hemoglobinase of erythrocytic *P. falciparum* trophozoites. Many *in vitro* studies have confirmed that inhibitors of falcipain-2 can block parasite hemoglobin hydrolysis and halt the development of culture parasites [8,9,10,11,12,13,14,15,16,17,18,19,20,21,22]. Some of them were also effective against murine malaria *in vivo* [15,21,22]. However, FP-2 inhibitors reported in the literature are mainly derived from peptide analogues [8,9,10,12,20], which tend to form covalent bonds with the thiolate of the catalytic cysteine and commonly have nanomolar IC_50_ values. Obviously, it is desirable to design non-peptidic inhibitors that would bind non-covalently to the target enzyme, in order to minimize toxicity while retaining the potential for high *in vivo* activity and selectivity. 

Recently, crystal structures for falcipain-2 have been reported [23,24], and the reservoir of structural and functional information of FP-2 has offered a solid starting point for the rational structure-based design of novel antimalarial drugs targeting FP-2. By using a docking-based virtual screening approach in conjunction with an enzyme inhibition assay, a novel small molecule inhibitor of FP-2 featuring the 2-(3,4-dihydro-4-oxothieno[2,3-*d*]pyrimidin-2-ylthio) acetamide framework (compound **1**) has been discovered [25]. After the identification of compound **1** as a possible prototype for the design of selective inhibitors of FP-2, fifteen new compounds, including **1** and its fourteen analogs (**2a**-**e**, **3a-c,** and **4a-f**), have been synthesized and tested against FP-2. All of the compounds show high inhibitory effect against FP-2 with IC_50_s of 1.46-11.38 μM, and inhibitory activity of compound **2a** increases ~2 times than that of prototype compound **1**. The preliminary SARs are summarized and should be helpful for future design of inhibitors.

## Results and Discussion

### Identification of Prototype (Hit) ***1*** by Virtual Screening

**Figure 1:**
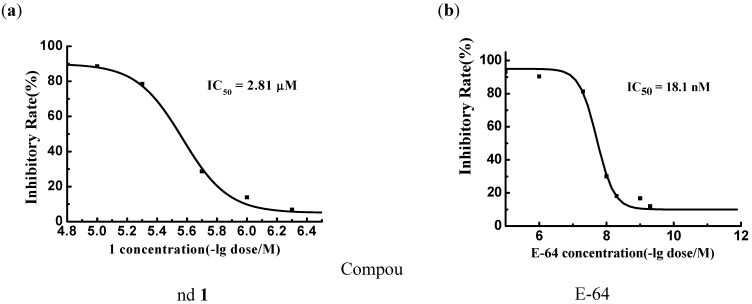
Concentration dependence of inhibitory activity by **1** and E-64, the concentration of FP-2 was kept constant at 30 nM while the concentration of compounds ranged from 0.001 to 10 μM.

Targeting the crystal structure of falcipain-2 (PDB entry 2GHU) [23], a total of 80,000 compounds were subsequently docked and ranked according to the software Glide and GAsDock [25]. Finally, 81 compounds were purchased and submitted to biological evaluations against falcipain-2. Among the 81 compounds, the inhibitory activity of compound **1** was concentration-dependent. The collected data indicated that compound **1** can inhibit FP-2 *in vitro*, with the inhibitory effect against FP-2 is in the micromolar range (IC_50_ = 2.81 μM). The substrate (L-3-*trans*-carboxyoxiran-2-carbonyl)-L-leucyl-agmatin (E-64), whose inhibitory effect against FP-2 is in the subnanomolar range (IC_50_ = 18.1 nM), was used for the positive control. Compound **1** could be designated as a hit of FP-2. The inhibitory rates (%) off compounds **1** and E-64 plotted against the common logarithm of the compound concentrations are shown in Figures 1a and 1b, respectively.

### Design and Synthesis of Compounds ***1-4***

On the basis of the framework of compound **1**, fourteen compounds (**2a**-**e**, **3a-c,** and **4a-f**) were designed and synthesized. Their chemical structures are shown in Table 1. Retaining the common 2-(3,4-dihydro-4-oxothieno[2,3-*d*]pyrimidin-2-ylthio) acetamide framework of compound **1**, we first changed the allyl group on the pyrimidine ring into cyclohexyl, aryl, and benzyl groups, and obtained analogs **2a-e** (Table 1). We then replaced the phenyl group on the thiophene ring with cyclohexyl or substituted phenyl groups to prepare compounds **3a-c** (Table 1). Finally, compounds **4a**-**f** (Table 1) were achieved by replacing the amide *N*-substituents with other aryl groups.

**Scheme 1:**
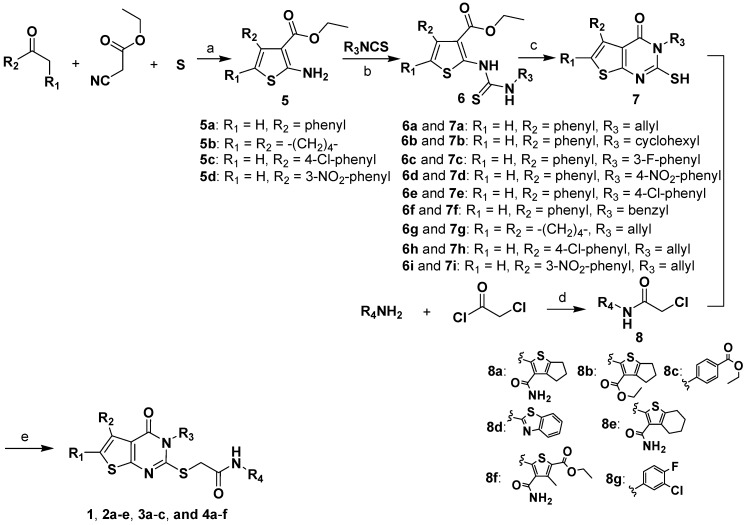
*^a^* The synthetic route to compounds **1**-**4**.

Compounds **1-4** were synthesized through the route outlined in Scheme 1. Using our previous method [26], using basic aluminium oxide as solid support and morpholine as base catalyst, ketones, cyanoacetates, and sulphur were mixed and subjected to microwave irradiation for several minutes, which afforded 2-aminothiophenes **5a-d**. Compounds **5a-d** were converted to the corresponding thioureas **6a-i** by reaction with isothiocyanates, then **6a-i** were cyclized under alkaline conditions, giving the key intermediates **7a-i** (Scheme 1). Various aromatic amines were chloracetylated with chloroacetic chloride to give compounds **8a-g**, which were reacted with compounds **7a-i** to produce the target compounds **1**-**4** smoothly. The details of the synthetic procedures and product characterizations are described in the Experimental section.

**Table 1:** Chemical Structures of Compounds **1**, **2a**-**e**, **3a**-**c** and **4a**-**f** and Their Inhibitory Activities against FP-2. 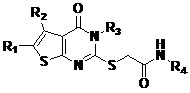

Compd.	R_1_	R_2_	R_3_	R_4_	Inhibition rate at 10 μM (%)	IC_50_ (μM)
**1**	H	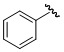	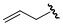	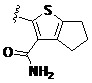	88.7	2.81
**2a**	H	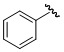	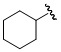	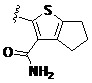	92.7	1.46
**2b**	H	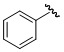	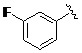	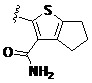	79.0	2.05
**2c**	H	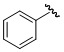	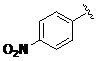	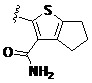	85.4	2.77
**2d**	H	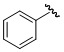	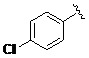	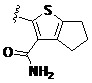	84.7	4.30
**2e**	H	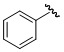	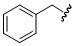	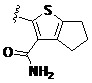	90.6	5.74
**3a**	—(CH_2_)_4_—		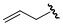	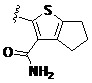	82.2	5.77
**3b**	H	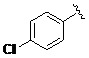	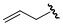	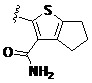	85.7	2.95
**3c**	H	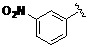	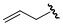	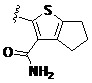	53.0	11.8
**4a**	H	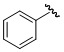	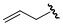	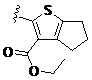	93.3	6.63
**4b**	H	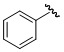	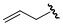	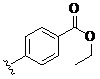	94.3	5.70
**4c**	H	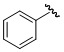	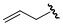	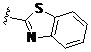	90.3	3.31
**4d**	H	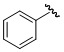	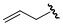	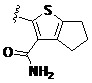	93.2	2.49
**4e**	H	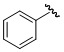	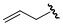	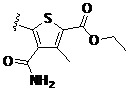	72.0	5.58
**4f**	H	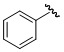	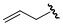	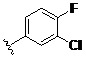	92.0	5.43

### Enzyme Inhibition Assay

For the primary assay, the percent inhibitions of the compounds at 10 μM were measured. The results are listed in Table 1. All compounds can remarkably inhibit the activity of FP-2 (Percent inhibition at 10 μM > 50%), indicating that these fifteen compounds (including compound **1**) are FP-2 inhibitors. Therefore, we determined their IC_50_ values (Table 1). From the data in Table 1, we can see that the percent inhibitory rates at 10 μM of compounds **2a**, **2e**, **4a**-**d**, and **4f** obviously increased (more than 90%, Table 1). Among five anologs varied on R_3_ substituents (**2a**-**2e**, Table 1), it is remarkable that the inhibitory activity of compound **2a** increased ~2 times than that of compound **1** (IC_50_ decreases from 2.81 μM down to 1.46 μM, Table 1), the former replace the allyl group on pyrimidine ring with a cyclohexyl group. The percent inhibitory rates at 10 μM of analogs **3a-c**, which vary on R_1_ and R_2_ substituents, were decreased in different extent, especially analogue **3c** which showed a marked decrease from 88.7% to 53.0% (Table 1). Changes of R_4_ substituents did not have a distinct improvement for the IC_50_s of analogs **4a**-**f** (Table 1).

*^a^* Reagents and conditions: (a) MWI, morpholine, basic Al_2_O_3_, 140 W, 15 min; (b) pyridine, 45 °C, 5 h; (c) CH_3_ONa, CH_3_OH, 25 °C, 10 h; (d) THF, Et_3_N, 25 °C, 10 h; (e) THF, K_2_CO_3_, 40 °C, 10 h.

### Structure and Activity Relationships

After testing the inhibitory activities of compounds **1**, **2a**-**e**, **3a-c,** and **4a-f** against FP-2, from the data in Table 1, we can deduce the preliminary structure-activity relationships (SARs) as follows: (1) Replacing the allyl group of compound **1** with proper substituent, such as a cyclohexyl (**2a**) group, moderately improves potency, as seen with the IC_50_ of compound **2a** which is decreased to 1.46 μM (Table 1); (2) Replacing the phenyl group on the thiophene ring of **1** with cyclohexyl (**3a**) or substituted phenyl (**3b**-**3c**) can not be tolerated and leads to the loss of inhibitory activity; (3) N-substituents of amide of compound **1** have considerable adaptability and alterability. This encouraging result and primary SARs are helpful for future inhibitors design.

## Conclusions

In this study, we have discovered a new kind of scaffold of FP-2 inhibitor by using a structure-based virtual screening approach in conjunction with chemical synthesis and bioassay. The preliminary SARs were obtained, which show changes of substituents on 3, 5, and 6-positions of thieno[2,3-*d*]pyrimidine moiety have very important influence on inhibitory activity, and appropriate structural optimizations on the above regions can substantially improve potency. These primary SARs are helpful for future inhibitors design, and the novel scaffold presented here with potent inhibitory activity against FP-2 also provides potential application in discovery of anti-malarial drugs.

## Experimental

Reagents were purchased from Alfa, Acros and Shanghai Chemical Reagent Company, and used without further purification. Analytical thin-layer chromatography (TLC) was performed on HSGF 254 plates (150-200 µm thickness, Yantai Huiyou Company, P.R. China). Yields were not optimized. Melting points were measured in capillary tube on a SGW X-4 melting point apparatus without correction. Nuclear magnetic resonance (NMR) spectra were given on a Bruker AVANCE 500 NMR (TMS as internal standard). Chemical shifts were reported in parts per million (ppm, *δ*) downfield from tetramethylsilane. Proton coupling patterns were described as singlet (s), doublet (d), triplet (t), quartet (q), multiplet (m), and broad (br). Low- and high-resolution mass spectra (LRMS and HRMS) were given with electrospray (ESI) produced by LCQ-TOF spectrometer. Microwave experiments were carried out in a domestic microwave oven (Haier MA-2270EGC)

### Virtual Screening by Molecular Docking

The crystal structure of falcipain-2 (PDB entry 2GHU) [23] from *Plasmodium falciparum* was retrieved from the Protein Database Bank [27]. Residues located within 14 Å from the catalytic thiolate of Cys42 were defined as part of the binding site for docking studies. All crystallographic water molecules were removed from the coordinate set. The pipeline of virtual screening based on molecular docking method is presented in detail in the reference 25.

### Enzyme Inhibition Assay

The purification and refolding of recombinant protein Falcipain-2 was performed as described by Shenai et al [6]. IC_50_ values against recombinant Falcipain-2 were determined as described previously [8,28]. FP-2 (30 nM) was incubated for 30 min at room temperature in 100 mM sodium acetate, pH 5.5, 10 mM DTT, with different concentrations of tested inhibitors. Inhibitor solutions were prepared from stock in DMSO (maximum concentration of DMSO in the assay was 1%). After 30 min incubation, the substrate Z-Leu-Arg-AMC (benzoxycarbonyl-Leu-Arg-7-amino-4-methylcoumarin, Bachem AG) in the same buffer was added to a final concentration of 25 μM. The increase in fluorescence (excitation at 355 nM and emission at 460 nM) was monitored for 30 min at room temperature with an automated microtiter plate spectrofluorimeter (Molecular Devices, Flex station). Half-maximal inhibitory concentration (IC_50_) was determined from plots of percent activity over compound concentration using GraphPad Prism software.

### General procedures for the preparation of 2-(3,4-dihydro-4-oxothieno[2,3-*d*]pyrimidin-2-ylthio)-acetamides ***1, 2a-e, 3a-c, and 4a-f,*** exemplified by the preparation of compound ***1***

*Ethyl 2-amino-4-phenylthiophene-3-carboxylate* (**5a**). A one-neck 50-mL flask containing acetophenone (2.4 g, 20 mmol), ethyl cyanoacetate (3.4 g, 30 mmol), sulphur (0.96 g, 30 mmol), basic Al_2_O_3_ (1.8 g), and morpholine (2.6 g, 30 mmol) was placed into a microwave oven and irradiated at the power of 140W for 15 min. After cooling, the residue was separated by column chromatography with silica gel using petroleum ether/ethyl acetate (10/1) as eluting solution, to afford **5a** (2.0 g, 27%) as a yellow solid: mp 86-90 °C; ^1^H-NMR (CDCl_3_) *δ* 0.92 (t, 3H), 4.03 (q, 2H), 6.06 (s, 1H), 7.30-7.32 (m, 5H). Compounds **5b**-**d** were prepared in a similar manner.

*3-Allyl-2-mercapto-5-phenylthieno[2,3-d]pyrimidin-4(3H)-one* (**7a**). A mixture of **5a** (1 g, 4 mmol) and allyl isocyanate (0.5 g, 5 mmol) in THF (5 mL) was stirred at 45 °C for 5 h. Concentration of the reaction mixture gave the compound **6a**, which was suspended in MeOH (5 mL). To this suspension was added MeONa (0.55 g, 10 mmol).After being stirred at room temperature for 10 h, the mixture was adjusted to pH 1 with 2 N HCl at 0 °C. The solution was extracted with EtOAc. The combined organic layer was washed, dried, filtered and concentrated. The residue was separated by column chromatography with silica gel using petroleum ether/ethyl acetate (5/1) as eluting solution to afford **7a** (0.26 g, 22%) as a yellow solid: mp 194-197 °C; ^1^H-NMR (CDCl_3_) *δ* 5.11 (d, 2H), 5.25 (d, 1H), 5.37 (d, 1H), 5.96-6.01 (m, 1H), 6.82 (s, 1H), 7.38-7.43 (m, 3H), 7.47-7.49 (m, 2H), 11.85 (s, 1H). Compounds **7b**-**i** were prepared in a similar manner.

*2-(2-Chloroacetamido)-5,6-dihydro-4H-cyclopenta[b]thiophene-3-carboxamide* (**8a**). To a stirred, room-temperature solution of 2-amino-5,6-dihydro-4*H*-cyclopenta[*b*]thiophene-3-carboxamide (180 mg, 1 mmol, which was prepared in the same manner as described for **5a**, triethylamine (0.1 mL) and THF (2 mL) was added dropwise chloroacetic chloride (170 mg, 1.5 mmol). After 10 hours, poured into H_2_O (10 mL), and extracted with EtOAc. The combined organic layer was washed, dried, filtered and condensed. The residue was purified by flash column chromatography on silica gel, eluted with a mixture of EtOAc/petroleum ether (1:2, v/v), to afford **8a** (129 mg, 50%) as white solid. ^1^H-NMR (CDCl_3_) *δ* 2.33-2.36 (m, 2H), 2.80 (t, 2H), 2.91 (t, 2H), 4.52 (s, 2H). Compounds **8b**-**g** were similarly prepared.

*2-(2-(3-Allyl-3,4-dihydro-4-oxo-5-phenylthieno[2,3-d]pyrimidin-2-ylthio)acetamido)-5,6-dihydro-4H-cyclopenta[b]thiophene-3-carboxamide* (**1**) A mixture of **7a** (132 mg, 0.44 mmol), **8a** (114 mg, 0.44 mmol), and K_2_CO_3_ (0.6 g, 4.4 mmol) in THF (5 mL) was stirred at 40 °C for 10 h. poured into H_2_O (10 mL), extracted with EtOAc. The combined organic layer was washed, dried, filtered and condensed. The residue was purified by flash column chromatography on silica gel, eluted with a mixture of EtOAc/petroleum ether (1:2, v/v), to afford **1** (68 mg, 30%) as yellow solid: mp 218-221 °C; ^1^H-NMR (DMSO) *δ* 2.32-2.36 (m, 2H), 2.76 (t, 2H), 2.89 (t, 2H), 4.26 (s, 2H), 4.74 (d, 2H), 5.17-5.25 (m, 2H), 5.91-5.97 (m, 1H), 7.34-7.39 (m, 4H), 7.47-7.49 (m, 2H); ESI-MS *m*/*z* 545 [M+Na]^+^; HRMS (ESI) m/z calcd C_25_H_22_N_4_O_3_S_3_Na [M+Na]^+^ 545.0752, found 545.0745. 

*2-(2-(3-Cyclohexyl-3,4-dihydro-4-oxo-5-phenylthieno[2,3-d]pyrimidin-2-ylthio)acetamido)-5,6- dihydro-4H-cyclopenta[b]thiophene-3-carboxamide* (**2a**) Replacing allyl isocyanate with cyclohexyl isocyanate, **2a** was prepared as a yellow solid: mp 225-229 °C; ^1^H-NMR (DMSO) *δ* 1.35 (m, 2H), 1.83 (m, 4H), 2.30-2.36 (m, 2H), 2.50-2.56 (m, 4H), 2.78 (t, 2H), 2.89 (t, 2H), 4.16 (m, 1H), 4.21 (s, 2H), 7.31 (s, 1H), 7.34-7.39 (m, 3H), 7.44 (m, 2H); ESI-MS *m*/*z* 565 [M+H]^+^; HRMS (ESI) m/z calcd C_28_H_29_N_4_O_3_S_3_ [M+H]^+^ 565.1402, found 565.1411.

*(2-(2-(3-(3-Fluorophenyl)-3,4-dihydro-4-oxo-5-phenylthieno[2,3-d]pyrimidin-2-ylthio)acetamido)-5,6-dihydro-4H-cyclopenta[b]thiophene-3-carboxamide* (**2b**) Replacing allyl isocyanate with 3-fluoro-phenyl isocyanate, **2b** was prepared as a pale yellow solid: mp 234-239 °C; ^1^H-NMR (DMSO) *δ* 2.31-2.36 (m, 2H), 2.78 (t, 2H), 2.90 (t, 2H), 4.10 (s, 2H), 7.30-7.35 (m, 2H), 7.43 (s, 1H), 7.46-7.48 (m, 4H), 7.58-7.68 (m, 3H); ESI-MS *m*/*z* 599 [M+Na]^+^; HRMS (ESI) m/z calcd C_28_H_21_N_4_O_3_FS_3_Na [M+Na]^+^ 599.0658, found 599.0667.

*(2-(2-(3-(4-Nitrophenyl)-3,4-dihydro-4-oxo-5-phenylthieno[2,3-d]pyrimidin-2-ylthio)acetamido)-5,6-dihydro-4H-cyclopenta[b]thiophene-3-carboxamide* (**2c**) Replacing allyl isocyanate with 4-nitrophenyl isocyanate, **2c** was prepared as an orange solid: mp 271-274 °C; ^1^H-NMR (CDCl_3_) *δ* 2.47-2.50 (m, 2H), 2.86-2.90 (m, 4H), 4.08 (s, 2H), 7.04 (s, 1H), 7.25-7.36 (m, 3H), 7.47-7.50 (m, 2H), 7.71 (d, 2H), 8.40 (d, 2H); ESI-MS *m*/*z* 626 [M+Na]^+^; HRMS (ESI) m/z calcd C_28_H_21_N_5_O_5_S_3_Na [M+Na]^+^ 626.0603, found 626.0605.

*(2-(2-(3-(4-Chlorophenyl)-3,4-dihydro-4-oxo-5-phenylthieno[2,3-d]pyrimidin-2-ylthio)acetamido)-5,6-dihydro-4H-cyclopenta[b]thiophene-3-carboxamide* (**2d**) Replacing allyl isocyanate with 4-chloro-phenyl isocyanate, **2d** was prepared as a yellow solid: mp 247-249 °C; ^1^H-NMR (CDCl_3_) *δ* 2.47-2.54 (m, 2H), 2.89-2.93 (m, 4H), 4.08 (s, 2H), 7.04 (s, 1H), 7.32-7.36 (m, 3H), 7.41-7.43 (m, 2H), 7.52 (d, 2H), 7.54 (d, 2H); ESI-MS *m*/*z* 615 [M+Na]^+^; HRMS (ESI) m/z calcd C_28_H_21_N_4_O_3_ClS_3_Na [M+Na]^+^ 615.0362, found 615.0351.

*(2-(2-(3-Benzyl-3,4-dihydro-4-oxo-5-phenylthieno[2,3-d]pyrimidin-2-ylthio)acetamido)-5,6-dihydro-4H-cyclopenta[b]thiophene-3-carboxamide* (**2e**) Replacing allyl isocyanate with benzyl isocyanate, **2e** was prepared as a gray solid: mp 163-167 °C; ^1^H-NMR (CDCl_3_) *δ* 2.48-2.53 (m, 2H), 2.89-2.96 (m, 4H), 4.16 (s, 2H), 4.26 (s, 2H), 7.00 (s, 1H), 7.30-7.43 (m, 8H), 7.54-7.56 (m, 2H); ESI-MS *m*/*z* 595 [M+Na]^+^; HRMS (ESI) m/z calcd C_29_H_24_N_4_O_3_S_3_Na [M+Na]^+^ 595.0908, found 595.0908.

*(2-(2-(3-Allyl-3,4,5,6,7,8-hexahydro-4-oxo-benzo[4,5]thieno[2,3-d]pyrimidin-2-ylthio) acetamido)-5,6-dihydro-4H-cyclopenta[b]thiophene-3-carboxamide* (**3a**) Replacing acetophenone with cyclo-hexanone, **3a** was prepared as a yellow solid: mp 247-250 °C; ^1^H-NMR (CDCl_3_) *δ* 1.82-1.87 (m, 4H), 2.48-2.52 (m, 2H), 2.71 (t, 2H), 2.88-2.91 (m, 4H), 2.97 (t, 2H), 4.15 (s, 2H), 4.83 (d, 2H), 5.31 (m, 2H), 5.92 (m, 1H); ESI-MS *m*/*z* 523 [M+Na]^+^; HRMS (ESI) m/z calcd C_23_H_24_N_4_O_3_S_3_Na [M+Na]^+^ 523.0908, found 523.0911.

*2-(2-(3-Allyl-3,4-dihydro-4-oxo-5-(4-chlorophenyl)thieno[2,3-d]pyrimidin-2-ylthio) acetamido)-5,6-dihydro-4H-cyclopenta[b]thiophene-3-carboxamide* (**3b**) Replacing acetophenone with 4-chloroacetophenone, **3b** was prepared as a gray solid: mp 236-239 °C; ^1^H-NMR (CDCl_3_) *δ* 2.49-2.54 (m, 2H), 2.89-2.93 (m, 4H), 4.20 (s, 2H), 4.83 (d, 2H), 5.28-5.35 (m, 2H), 5.90-5.98 (m, 1H), 6.97 (s, 1H), 7.38 (d, 2H), 7.47 (d, 2H); ESI-MS *m*/*z* 579 [M+Na]^+^; HRMS (ESI) m/z calcd C_25_H_21_N_4_O_3_S_3_ClNa [M+Na]^+^ 579.0362, found 579.0350.

*2-(2-(3-Allyl-3,4-dihydro-4-oxo-5-(3-nitrophenyl)thieno[2,3-d]pyrimidin-2-ylthio)acetamido)-5,6-dihydro-4H-cyclopenta[b]thiophene-3-carboxamide* (**3c**) Replacing acetophenone with 3-nitroacetophenone, **3c** was prepared as a yellow solid: mp 157-161 °C; ^1^H-NMR (CDCl_3_) *δ* 2.47-2.51 (m, 2H), 2.87-2.91 (m, 4H), 4.19 (s, 2H), 4.83 (d, 2H), 5.26-5.31 (m, 2H), 5.88-5.97 (m, 1H), 7.08 (s, 1H), 7.56 (q, 1H), 7.88 (d, 1H), 8.20 (d, 1H), 8.37 (s, 1H); ESI-MS *m*/*z* 590 [M+Na]^+^; HRMS (ESI) m/z calcd C_25_H_21_N_5_O_5_S_3_Na [M+Na]^+^ 590.0603, found 590.0616.

*Ethyl 2-(2-(3-allyl-3,4-dihydro-4-oxo-5-phenylthieno[2,3-d]pyrimidin-2-ylthio)acetamido)-5,6-dihydro-4H-cyclopenta[b]thiophene-3-carboxylate* (**4a**) Replacing 2-amino-5,6-dihydro-4*H*-cyclopenta[*b*]thiophene-3-carboxamide with ethyl 2-amino-5,6-dihydro-4*H*-cyclopenta[*b*]thiophene-3-carboxylate, **4a** was prepared as a white solid: mp 166-170 °C; ^1^H-NMR (DMSO) *δ* 1.28 (t, 3H), 2.28-2.33 (m, 2H), 2.77-2.83 (m, 4H), 4.28 (q, 2H), 4.32 (s, 2H), 4.73 (d, 2H), 5.20-5.24 (m, 2H), 5.90-5.97 (m, 1H), 7.20 (s, 1H), 7.35-7.38 (m, 3H), 7.46-7.48 (m, 2H); ESI-MS *m*/*z* 574 [M+Na]^+^; HRMS (ESI) m/z calcd C_27_H_25_N_3_O_4_S_3_Na [M+Na]^+^ 574.0905, found 574.0907.

*Ethyl 4-(2-(3-allyl-3,4-dihydro-4-oxo-5-phenylthieno[2,3-d]pyrimidin-2-ylthio)acetamido) benzoate* (**4b**) Replacing 2-amino-5,6-dihydro-4*H*-cyclopenta[*b*]thiophene-3-carboxamide with ethyl 4-amino-benzoate, **4b** was prepared as a white solid: mp 162-164 °C; ^1^H-NMR (CDCl_3_) *δ* 1.40 (t, 3H), 4.05 (s, 2H), 4.37 (q, 2H), 4.79 (d, 2H), 5.30-5.35 (m, 2H), 5.86-5.96 (m, 1H), 7.09 (s, 1H), 7.41-7.45 (m, 3H), 7.54-7.57 (m, 2H), 7.62 (d, 2H), 8.02 (d, 2H); ESI-MS *m*/*z* 528 [M+Na]^+^; HRMS (ESI) m/z calcd C_26_H_23_N_3_O_4_S_2_Na [M+Na]^+^ 528.1028, found 528.1033.

*2-(3-Allyl-3,4-dihydro-4-oxo-5-phenylthieno[2,3-d]pyrimidin-2-ylthio)-N-(benzo[d]thiazol-2-yl)-acetamide* (**4c**) Replacing 2-amino-5,6-dihydro-4*H*-cyclopenta[*b*]thiophene-3-carboxamide with benzo[*d*]thiazol-2-amine, **4c** was prepared as a white solid: mp 192-194 °C; ^1^H-NMR (CDCl_3_) *δ* 4.20 (s, 2H), 4.79 (d, 2H), 5.31-5.35 (m, 2H), 5.86-5.96 (m, 1H), 7.07 (s, 1H), 7.33-7.48 (m, 5H), 7.53-7.55 (m, 2H), 7.80-7.85 (m, 2H); ESI-MS *m*/*z* 513 [M+Na]^+^; HRMS (ESI) m/z calcd C_24_H_18_N_4_O_2_S_3_Na [M+Na]^+^ 513.0490, found 513.0502. 

*2-(2-(3-Alyl-3,4-dihydro-4-oxo-5-phenylthieno[2,3-d]pyrimidin-2-ylthio)acetamido)-4,5,6,7-tetra-hydrobenzo[b]thiophene-3-carboxamide* (**4d**) Replacing 2-amino-5,6-dihydro-4*H*-cyclopenta[*b*]-thiophene-3-carboxamide with 2-amino-4,5,6,7-tetrahydrobenzo[*b*]thiophene-3-carboxamide, **4d** was prepared as a white solid: mp 212-214 °C; ^1^H-NMR (CDCl_3_) *δ* 1.86 (m, 4H), 2.71 (m, 4H), 4.20 (s, 2H), 4.84 (d, 2H), 5.27-5.37 (m, 2H), 5.90-6.02 (m, 1H), 6.97 (s, 1H), 7.38-7.43 (m, 3H), 7.52-7.54 (m, 2H); ESI-MS *m*/*z* 559 [M+Na]^+^; HRMS (ESI) m/z calcd C_26_H_24_N_4_O_3_S_3_Na [M+Na]^+^ 559.0908, found 559.0915.

*Ethyl 5-(2-(3-allyl-3,4-dihydro-4-oxo-5-phenylthieno[2,3-d]pyrimidin-2-ylthio)acetamido)-4-carbamoyl-3-methylthiophene-2-carboxylate* (**4e**) Replacing 2-amino-5,6-dihydro-4*H*-cyclopenta[*b*]-thiophene-3-carboxamide with ethyl 5-amino-4-carbamoyl-3-methylthiophene-2-carboxylate, **4e** was prepared as a white solid: mp 136-140 °C; ^1^H-NMR (CDCl_3_) *δ* 1.40 (t, 3H), 2.80 (s, 3H), 4.19 (s, 2H), 4.40 (q, 2H), 4.80 (d, 2H), 5.27-5.33 (m, 2H), 5.86-5.94 (m, 1H), 6.96 (s, 1H), 7.36-7.43 (m, 3H), 7.49-7.52 (m, 2H); ESI-MS *m*/*z* 591 [M+Na]^+^; HRMS (ESI) m/z calcd C_26_H_24_N_4_O_5_S_3_Na [M+Na]^+^ 591.0807, found 591.0809.

*2-(3-Allyl-3,4-dihydro-4-oxo-5-phenylthieno[2,3-d]pyrimidin-2-ylthio)-N-(3-chloro-4-fluorophenyl)-acetamide* (**4f**) Replacing 2-amino-5,6-dihydro-4*H*-cyclopenta[*b*]thiophene-3-carboxamide with 3-chloro-4-fluorobenzenamine, **4f** was prepared as a yellow solid: mp 145-149 °C; ^1^H-NMR (CDCl_3_) *δ* 4.02 (s, 2H), 4.78 (d, 2H), 5.30-5.35 (m, 2H), 5.86-5.95 (m, 1H), 7.07-7.12 (m, 2H), 7.31-7.34 (m, 1H), 7.41-7.47 (m, 3H), 7.54-7.56 (m, 2H), 7.78 (q, 1H); ESI-MS *m*/*z* 508 [M+Na]^+^; HRMS (ESI) m/z calcd C_23_H_17_N_3_O_2_FClS_2_Na [M+Na]^+^ 508.0332, found 508.0328.
